# Technological Innovations to Support Family Caregivers: A Scoping Review

**DOI:** 10.3390/healthcare12232350

**Published:** 2024-11-25

**Authors:** Laura Fernandez-Bueno, Dolores Torres-Enamorado, Ana Bravo-Vazquez, Cleofas Rodriguez-Blanco, Carlos Bernal-Utrera

**Affiliations:** 1 Doctoral Program in Health Sciences, University of Seville, 41009 Seville, Spain; laurafernandez0895@gmail.com (L.F.-B.);; 2 Nursing Department, University of San Juan de Dios (Bormujos), 41009 Seville, Spain; 3 Nursing Department, Faculty of Nursing, Physiotherapy and Podiatry, University of Seville, 41009 Sevilla, Spain; 4 School of Nursing and Physiotherapy San Juan de Dios, Pontifical Comillas University, San Juan de Dios Foundation, 28036 Madrid, Spain; 5 Critical Care Unit, Traumatology and Rehabilitation, University Hospital Virgen del Rocío, 41013 Seville, Spain; 6 Physiotherapy Department, Faculty of Nursing, Physiotherapy and Podiatry, University of Seville, 41009 Sevilla, Spain; cleofas@us.es (C.R.-B.); cbutrera@us.es (C.B.-U.)

**Keywords:** caregivers, telemedicine, primary care nursing, eHealth strategies, nurse

## Abstract

Introduction: Population aging increases the risk of dependency among older adults, which in turn necessitates care, primarily provided by family caregivers. This situation leads to physical and emotional strain on these caregivers. New technologies, such as tele-education, digital platforms, or mobile applications, can offer an accessible and equitable alternative for caregiver training and self-care support. Objective: The objective of this review is to analyze interventions targeted at family caregivers, both for their own self-care and for the care of dependent individuals, using new technologies. Design: A scoping review was conducted, including a total of thirty-two articles extracted from three databases: CINAHL, Scopus, and PubMed. Articles in any language were included, with no fixed time limit, while articles with samples that included family caregivers of oncology patients were excluded. Results: Most of the interventions were conducted via videoconference, showing outcomes that indicated a reduction in depressive symptoms among family caregivers. Conclusions: The implementation of new technologies for the development of interventions presents a viable alternative to in-person sessions. These technologies have shown positive results, while also helping to overcome time and geographical barriers imposed by caregiving responsibilities.

## 1. Introduction

The aging of the global population is currently considered one of the main challenges to address. According to estimates by the World Health Organization, by 2030, one-sixth of the world’s population will be 60 years or older. Further statistical projections indicate that by 2050, this number is expected to double [1].

Population aging not only reshapes global social structures but also carries significant economic, social, and political consequences. The increase in life expectancy in the global population is not linked to an improvement in the quality of life of older adults, as there has not been a corresponding enhancement in lifestyle habits. As a result, chronic diseases and their potential consequences, such as acquired dependency due to chronic illnesses or disabilities, become more pronounced. These conditions often develop gradually and may require long-term care. The growing number of older adults with dependencies has led to an increasing demand for caregivers to address their needs for assistance. In most cases, these caregivers are women from the family environment, who, without receiving any form of compensation, take on the responsibility of providing for the basic and instrumental needs that the dependent individual cannot manage themselves. These individuals are referred to as family caregivers or informal caregivers. While women make up the majority of caregivers, the term ‘family caregivers’ encompasses all individuals, regardless of gender, who take on the role of caring for a relative in a state of dependency [2].

The caregiving situation can evolve gradually or, in some cases, emerge abruptly, depending on the condition of the person receiving care. The challenges of caring for someone with cognitive decline can differ from those of caring for an individual with a progressive chronic illness. In either scenario, caregiving places an increasing physical and psychological burden on the caregiver, which can ultimately affect their health. The first year of caregiving is often marked by the most significant changes in all aspects of the caregiver’s life, particularly in physical and emotional health, but also in social, professional, familial, and financial domains [3].

Currently, healthcare and social services are unable to meet the high demand for care required by the dependent population, making the role of the family caregiver crucial. The care they provide has a profound impact on their own lives, significantly increasing the risk of burnout and often turning them into secondary or even invisible patients. We refer to family caregivers as “invisible patients” or “hidden patients” because, by dedicating a large part of their time and effort to caring for a dependent loved one, they tend to neglect their own physical and emotional health. The consequences of being a hidden patient are both significant and concerning. The lack of attention to their own health can make these caregivers more vulnerable to illness, eventually turning them into patients themselves. Therefore, it is crucial to recognize and address the health needs of caregivers to prevent their vital role in caring for dependents from negatively impacting their well-being [4].

Caregivers face numerous challenges in adapting to the schedules of in-person health programs due to time constraints, conflicting dates, or schedule incompatibilities caused by work, geographical barriers, additional family caregiving responsibilities, or other circumstances like transporting the dependent person to day centers or medical appointments [4,5]. This situation highlights a systemic inequality in healthcare, where caregiving responsibilities are still closely tied to gender roles, with the expectation that women should provide this care [6]. Since the 1990s, telephone support groups have been introduced as an alternative to replace in-person meetings, but this option continues to limit the exchange of information, as it excludes non-verbal communication and the ability to demonstrate specific techniques or postures. However, technological advancements over the past 30 years have enabled the inclusion of video-based training, both synchronously and asynchronously, which helps overcome scheduling conflicts and geographical barriers while improving information retention. These technological interventions aimed at caregivers range from synchronous communication platforms based on video or text, allowing real-time interaction; online health education classes; and even peer support forums, where caregivers can share experiences and receive professional guidance. On the other hand, asynchronous support includes tools such as discussion forums, group communication via email, digital libraries with educational materials, informational videos, and access to updated clinical reports. Additionally, group interventions hosted on social media platforms, such as Facebook, have been evaluated in recent studies, showing positive results and potential in the context of caregiver support [5]. This tele-education could be an alternative for these caregivers, incorporating telehealth training.

Support for caregivers would improve by promoting equitable and accessible care, regardless of the caregiver’s location, whether in a rural or urban area, their level of technological literacy, health knowledge, socioeconomic status, or even age. Such advancements would ensure access to high-quality care [7]. However, it is necessary to explore what is available in the literature regarding the different interventions that have been carried out so far using new technologies and their effectiveness.

Therefore, the objective of this scoping review is to identify and analyze interventions aimed at family caregivers, focusing on both for their self-care and the care of dependent individuals, through the use of new technologies.

## 2. Materials and Methods

This review was conducted in accordance with the guidelines of the Joanna Briggs Institute (JBI) and followed the methodological framework first published in 2005 by Arksey and O’Malley, which involves completing five stages: (1) identifying the research question, (2) searching for relevant studies, (3) selecting studies, (4) data recording, and finally, (5) collating, summarizing, and reporting the results. In addition, to enhance the quality and transparency of the review, the PRISMA-ScR checklist was applied, it will be consulted in Supplementary Material S1. The protocol for this review was registered in the Open Science Framework (https://osf.io/hupej/, accessed on 31 January 2024) under the registration number DOI: https://doi.org/10.17605/OSF.IO/HUPEJ.

### 2.1. Identifying the Research Question

The primary question this scoping review aimed to answer is as follows: “What interventions targeting informal caregivers of dependent adults are being implemented using new technologies?”. The following secondary questions were also addressed:

– What new methods of intervention for caregivers are being implemented?
– Who are these interventions targeting?
– What interventions are focused on caregivers’ health?
– Is any type of follow-up conducted after these interventions?
– What outcomes have been achieved from the interventions carried out?

The eligibility of the studies for the main research question was defined using the Participant, Concept, and Context (PCC) framework: the population was family caregivers of dependent adults, the concept focused on interventions carried out using new technologies, and no restrictions were imposed regarding the context [8].

### 2.2. Identifying Relevant Studies

#### 2.2.1. Eligibility Criteria

Studies involving informal caregivers of dependent older adults were included. The review considered a variety of study designs, including cross-sectional, retrospective cohort, prospective, and randomized controlled trials. This scope included studies in any language and with no fixed time limit. However, studies where the population consisted of informal caregivers of cancer patients were excluded, as the interventions for these caregivers have a different focus. Caregivers of oncology patients were excluded, since the needs and intervention objectives for this population often differ significantly from those aimed at caregivers of individuals with chronic or acute illnesses. Family caregivers of oncology patients face specific challenges related to pain management, communication about treatment, and emotional support in an intensive and often changing treatment context.

On the other hand, family caregivers of individuals with chronic or acute conditions tend to deal with a different set of circumstances, including managing multiple comorbidities, long-term care, and emotional exhaustion resulting from prolonged dependency.

By focusing on caregivers of individuals with chronic or acute conditions, we aim to identify interventions that are relevant and effective for a population that shares similar characteristics and needs.

#### 2.2.2. Information Sources

Advanced searches were conducted in the electronic databases Cumulative Index of Nursing and Allied Health Literature (CINAHL), PubMed, and Scopus. The literature search was conducted by the authors from 1 November 2023 to 30 January 2024.

#### 2.2.3. Search Strategies

The search was conducted using a combination of MeSH terms (Medical Subject Headings) and keywords such as “family caregivers” AND “health education” AND “eHealth strategies”, developed by all the authors. The search string can be seen in Table 1.

### 2.3. Study Selection

**Figure 1:**
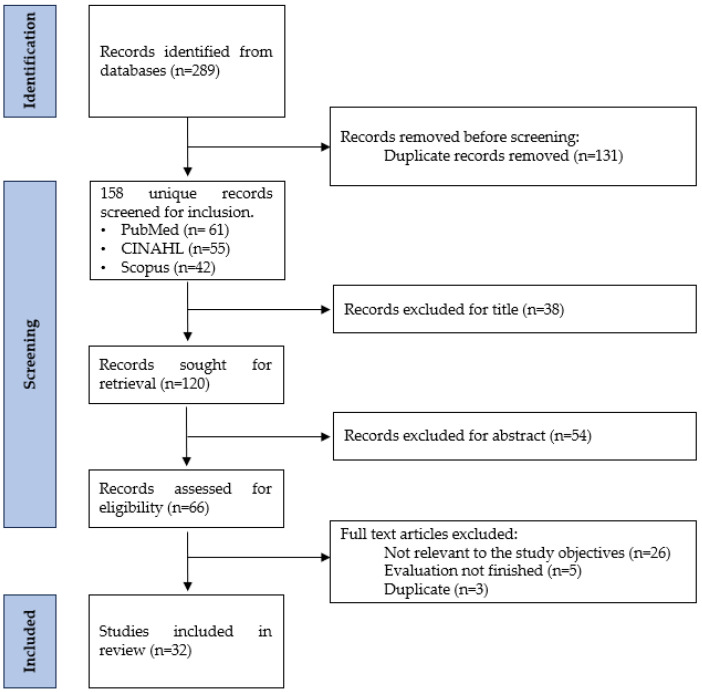
PRISMA flow diagram (Preferred Reporting Items for Systematic Reviews and Meta-Analyses) 2020 [9].

A total of 289 articles were initially identified. The search results were exported to the reference manager Mendeley Desktop (version 1.19.4), which was used to identify and remove duplicate articles. After excluding duplicates, 158 articles remained: 42 from Scopus, 61 from PubMed, and 55 from CINAHL. Subsequently, based on the pre-established eligibility criteria, the articles were screened by title and abstract, and the full text of those meeting the inclusion criteria was retrieved and evaluated. After this review, a total of 92 articles were excluded based on their title and abstract, leaving 66 articles. Following a full-text review, 34 additional articles were excluded: 26 did not meet the inclusion criteria for our study, 5 did not report project results because they had not been completed, and 3 were duplicates. As a result, 32 articles were finally included in the scoping review: 8 from CINAHL, 9 from Scopus, and 15 from PubMed. Figure 1 shows the data collection, selection, and extraction process used.

### 2.4. Charting the Data

#### 2.4.1. Data Extraction

The data were extracted and recorded in a spreadsheet developed and verified by reviewers.

#### 2.4.2. Data Charting

The extracted data include (a) study and participant characteristics, (b) characteristics of the interventions, (c) study outcomes, (d) year of publication, and (e) the country where the intervention took place. The types of interventions, their target audience, and the inclusion criteria were analyzed to describe the different interventions that applied to the study participants.

### 2.5. Collating, Summarizing, and Reporting Results

Based on the findings obtained from mapping the concepts underlying the main research question, a generalized overview of the subject was established, considering the quality of each study. A narrative synthesis of the data was also performed, using the information extracted from the data sheet. All the authors of this review discussed and agreed upon the final report of the results.

## 3. Results

The articles included in the review (n = 32) are presented in Table 2, where all the data obtained in the review can be observed.

### 3.1. General Information Regarding the Studies

#### 3.1.1. Year of Publication and Country of Study Conduct

Among the thirty-two articles included in this review, 12.5% of them were published in 2018 (four out of thirty-two), 15.65% of the articles (five out of thirty-two) in 2019, 12.5% of the articles (four out of thirty-two) in 2020, 6.25% of the articles (two out of thirty-two) in 2021, 28.13% in 2022 (nine out of thirty-two), 21.88% in 2023 (seven out of thirty-two articles), and 3.13% (one out of thirty-two) in January 2024. These studies were mostly conducted in the United States, accounting for 62.5% of the articles published (twenty out of thirty-two articles), 28.13% of the articles (nine out of thirty-two) in Europe, 9.38% (three out of thirty-two) in Asia and 3.13% in Australia (one out of thirty-two). Table 3 shows the different studies based on the country of publication and the interventions carried out. Upon analyzing the aforementioned table, we observe that 93.75% of the articles are published in developed countries, while only 6.25% have been published and conducted in developing countries.

#### 3.1.2. Participants and Sample Size

The participants in the reviewed articles were predominantly female family caregivers, with the percentage of female participants in each study ranging from 42.85% to 100%, with an average of 72.21%. The sample size of the reviewed articles mostly ranged from six to two-hundred and sixty-one participants.

#### 3.1.3. Methodology Carried out

We found different types of study designs. The most common design, appearing in 43.75% (fourteen out of thirty-two) of the studies, was the randomized controlled trial (RCT), followed by qualitative studies at 25% (eight out of thirty-two), descriptive studies at 12.5% (four out of thirty-two), mixed methods and non-randomized controlled trials, respectively, at 6.25% (two out of thirty-two), and observational studies and group interventions at 3.13% (one out of thirty-two), respectively.

#### 3.1.4. Intervention Duration

The different intervention periods ranged from 1 day (3.13%; one out of thirty-two) to 1 year (3.13%; one out of thirty-two). Most interventions lasted 8 weeks (25%; eight out of thirty-two), followed by 3 months (15.63%; five out of thirty-two), 4 weeks (12.5%; four out of thirty-two), 6 months (9.38%; three out of thirty-two), 6 weeks (6.25%; two out of thirty-two), 4 months (6.25%; two out of thirty-two), 3 days (3.13%; one out of thirty-two), 5 days (3.13%; one out of thirty-two), 2 weeks (3.13%; one out of thirty-two), 10 weeks (3.13%; one out of thirty-two), 5 months (3.13%; one out of thirty-two), and 9 months (3.13%; one out of thirty-two). All of this can be seen in Table 4.

### 3.2. Results of the Interventions

#### 3.2.1. Type of Intervention Conducted

The different studies conducted various types of interventions, all of which were completed. Firstly, we find that 71.88% of the reviewed articles featured interventions consisting of online video conference sessions (23 out of 32). Notably, 13.04% (three out of twenty-three) of these focused on tele-rehabilitation, 4.35% (one out of twenty-three) on online yoga, and 8.7% (two out of twenty-three) on tele-nursing, which involved comprehensive caregiver assessments along with health education sessions.

In some cases, 13.04% of these sessions were complemented by telephone sessions (three out of twenty-three), 4.35% by both telephone and in-person sessions (one out of twenty-three), 4.35% by personalized video recordings (one out of twenty-three), or by viewing videos and other psychoeducational materials in 8.7% (two out of twenty-three).

Other articles, 3.13%, involved in-person and telephone sessions (one out of thirty-two), or 6.25% of the total conducted only telephone training sessions (two out of thirty-two). In 3.13%, individual video recording sessions at home were carried out (one out of thirty-two).

Additionally, some articles, 3.13%, developed their own online platforms for video calls, video viewing, and other resources (one out of thirty-two), or 9.38% created an app with various resources and materials (three out of thirty-two).

Lastly, one study, representing 3.13% of our sample, conducted video consultation sessions (one out of thirty-two).

#### 3.2.2. Objective of the Interventions

In the reviewed articles, we found differences in the objectives of the various programs. Notably, 46.88% (fifteen out of thirty-two) of the articles aimed to improve the self-care of family caregivers, 21.88% (seven out of thirty-two) were directed at both the family caregiver and the care recipient, and 31.25% (ten out of thirty-two) were focused on improving the care provided to the care recipient by the caregiver.

#### 3.2.3. Development of the Intervention

We found articles that included follow-up with the intervention recipients (84.36%; twenty-seven out of thirty-two), while other articles did not show that any follow-up was conducted (15.63%; five out of thirty-two).

The follow-up of the intervention was carried out in different ways: some articles conducted follow-ups through pre- and post-intervention evaluations (9.38%; three out of thirty-two), or through follow-up after the intervention (28.13%; nine out of thirty-two), ranging from 1 week to 9 months after the intervention. Other articles performed follow-ups during the intervention (25%; eight out of thirty-two), weekly (15.63%; five out of thirty-two), every 2 weeks (3.13%; one out of thirty-two), between the first month and up to a year after the start of the intervention (12.5%; four out of thirty-two), or daily (9.38%; three out of thirty-two).

#### 3.2.4. Participant Profile

- The participants included in the articles are categorized based on the pathology of the care recipient:
- Family caregivers of people with dementia (S1, S3, S4, S8, S9, S12, S14, S18, S19, S21, S22, S25, S28, S29, S31). Notably, article S3 includes caregivers who must have provided care at least three times a week and experienced increased stress due to caregiving, while article S4 requires the sample to provide care to someone over 60 years old with a dementia diagnosis and behavioral and psychological symptoms. Both articles S8 and S12 also include the care recipient.
- Family caregivers of people with stroke (CVA) and their care recipients (S2, S26).
- Family caregivers of chronically ill older adults (S5), where the inclusion criterion is that caregivers have been unable to attend in-person sessions due to scheduling conflicts.
- Family caregivers of people with Congestive Heart Failure (S6, S17, S30).
- Family caregivers of people with diagnosed Alzheimer’s disease (S7, S12, S15, S24, S32). In article S7, caregivers also include users with other forms of dementia, and article S12 also includes the care recipient.
- Family caregivers of patients with hip fractures (S23, S27).
- Family caregivers of COVID-19 patients (S10).
- Family caregivers of people with advanced Parkinson’s disease (S11).
- Family caregivers of people with ALS (Amyotrophic Lateral Sclerosis) (S13).
- Family caregivers of people with spinal cord injuries and chronic neuropathic pain (S16).
- Family caregivers of patients undergoing hematopoietic stem cell transplantation (HCT) (S20).

#### 3.2.5. Results of the Interventions Conducted

If we analyze the impact of the different interventions on the study population, we observe a reduction in depressive symptoms (S1, S3, S7, S16, S19, S26), stress and anxiety (S1, S4, S15, S16, S26, S27), and caregiver role overload (S1, S2, S3, S10, S19, S21, S24, S32). This leads to an increase in self-compassion (S1, S21), improved psychosocial well-being (S14), and improved decision-making and emotional management in difficult situations (S1 and S21).

It is noteworthy that caregivers have improved their competencies, increasing their knowledge in managing situations and the specific illness of the care recipient (S2, S3, S5, S7, S10, S13, S14, S19, S21, S24, S25, S29), increased their interaction with other caregivers (S3, S5, S12, S15), learned relaxation and self-care techniques (S4, S5, S20), and improved sleep quality (S4, S19).

Regarding the care recipient, improvements included better outcomes for stroke patients (S2), increased protein intake not only for the care recipient but also for the caregiver (S8), reduced desire for institutionalization (S19), improved home recovery for the care recipient (S27), relevant information on recovery, prevention, and personalized exercises directed at both the care recipient and the family caregiver (S23), and improved effective communication between the caregiver and the care recipient (S9, S14, S17).

Regarding the use of new technologies for developing interventions, issues were encountered with connectivity (S6, S22, S25, S30), feasibility of online intervention (S4, S16, S28), and comfort and follow-up of sessions (S12). Age-related differences in the effectiveness of new technologies were also observed (S2, S18), with some participants preferring in-person sessions due to unfamiliarity with technology (S14). However, there is satisfaction among those who used these new technologies, such as through the use of an app (S20, S22, S23, S30), and a preference for online strategies (S24), which leads to economic savings (S32).

Finally, there is an institutional-level result, as we see an improvement in the nurse–family relationship, with the nurse gaining a better holistic view of the family (S17).

It is also important to note that some results showed no improvement with the use of new technologies for interventions (S8, S11, S13, S15, S18, S31).

## 4. Discussion

This scoping review evaluated various interventions, outcomes, participants, durations, and follow-up approaches aimed at informal caregivers of dependent adults, implemented through new technologies. The findings provide valuable insights for future research, enhancing the potential of these technologies to offer more effective support to family caregivers.

Among the 32 articles included in this review, a significant majority of participants were women, confirming that women continue to be the primary providers of family care. However, there is also evidence of a gradual increase in male participation in these roles, reflecting the current caregiving crisis [42]. Although this transition towards a more equitable distribution of caregiving responsibilities is progressing slowly, a gradual change is underway [43,44,45].

The variability in sample size (ranging from six to two-hundred and sixty-one participants) suggests a diversity in methodological approaches, but it may also indicate limitations in the generalizability of the results. The average percentage of female participants (72.21%) highlights the need for policies supporting women in these roles, given the significant impact on their health and well-being. An example of such policies is the European Care Strategy for caregivers and care recipients (2022), which reveals that 7.7 million women have left their jobs due to caregiving responsibilities. In response, the European Commission has proposed specific actions to improve working conditions and facilitate the reconciliation of family and professional life, as well as to promote training, counseling, and financial and psychological support [46].

The chronological analysis reveals a growing interest in this research field, particularly in 2022 and 2023, with 28.13% and 21.88% of studies published, respectively. This increase may be linked to the growing global concern about the mental health and well-being of family caregivers, possibly exacerbated by the COVID-19 pandemic, which has highlighted the importance of caregivers in the healthcare system. It is noteworthy that most studies were conducted in the United States (62.5%), which might reflect greater investment in research and resources in this context, though it also suggests a need to diversify study locations to capture cultural and contextual variations in caregivers’ experiences. Additionally, several studies conducted during the COVID-19 pandemic have reported positive results related to interventions based on new technologies [47,48,49,50].

The duration of interventions varied considerably, from one day to one year, reflecting the diversity of strategies and objectives. Longer interventions (8 weeks or more) appear to be more common (25%), possibly because behavioral and attitudinal changes in caregivers are expected to occur gradually. However, the effectiveness of shorter interventions also warrants attention, especially in contexts where caregivers may face time constraints.

A key finding from this review is that most interventions (71.88%) were conducted via online videoconference sessions. These sessions were effective in reducing caregiver role overload and improving depressive symptoms, while also removing barriers such as transportation difficulties or the inability to attend sessions at specific times [7]. However, these online sessions are intended to complement rather than replace in-person sessions for those who can attend. Additionally, several studies reported favorable outcomes from combining online and in-person sessions [51].

Despite these positive results, challenges related to technology adoption among older adults persist. Some studies indicate that a significant portion of both family caregivers and healthcare professionals have low interest in using new technologies among older age groups [52]. To address this issue, follow-up sessions can help resolve technological difficulties that may arise during the intervention.

Most interventions focused on caregiver self-care (46.88%), reflecting an emphasis on improving resilience and caregivers’ ability to manage stress. However, a considerable proportion also aimed at improving the quality of care provided, suggesting a recognition of the interdependence between caregiver well-being and the care they provide. This dual focus is crucial, as improving caregiver self-care could lead to better outcomes for the care recipient. For example, teleassistance can enhance home-based care, improve home safety, and provide guidance on accident prevention, which alleviates caregiver concern and helps monitor their health [53].

On the other hand, it is important to highlight that, although these interventions were initially aimed at caregiver self-care, they often include training for caring for the person requiring care, not exclusively for caregiver self-care.

Follow-up of interventions was common in most studies (84.38%), indicating an interest in assessing the sustained effects of interventions. The various follow-up approaches, from pre- and post-intervention evaluations to long-term follow-ups, reflect a commitment to the ongoing assessment of effectiveness, but also suggest a need to standardize follow-up practices to improve comparability between studies. Some authors emphasize the importance of systematic and personalized follow-ups to empower users, whether family caregivers or patients, to address challenges related to using new technologies [54]. This follow-up helps refine the use of these technologies by addressing barriers that may arise over time.

The diversity in inclusion criteria, ranging from caregivers of individuals with dementia (46.88%) to those caring for patients with conditions like Parkinson’s and ALS, shows the broad range of scenarios faced by family caregivers. This diversity suggests the need for personalized interventions tailored to specific needs of caregivers, based on the condition of the person receiving care. An example is a study that developed a digital platform for caregivers, providing information on the diet of care recipients with dysphagia, improving nutrition and reducing hospitalization risk for the care recipient [55]. Each situation and need must be considered independently, and interventions should be personalized based on both the care recipient’s and caregiver’s needs. The high number of studies including caregivers of individuals with dementia may be due to their high caregiver role overload, which focuses interventions on this group [56].

The most consistent outcomes were reductions in depressive symptoms, stress, and anxiety, suggesting that interventions are achieving their primary goal of improving caregivers’ mental health. However, the lack of improvement in some studies using new technologies underscores the importance of considering caregivers’ preferences and technological capabilities. Additionally, satisfaction with digital interventions and associated cost savings may encourage the adoption of these tools, provided that technological barriers are addressed.

However, our scoping review presents some limitations. First, the lack of a standardized assessment for all family caregivers may have led to differences in the results of the various articles, with varying reductions in depressive symptoms, anxiety, and stress. Additionally, the differences in sample size across studies, along with the heterogeneity in study design, may limit the generalization of the findings. Not all the reviewed articles provided sufficient data on the characteristics of the caregivers, their age, the relationship with the cared-for individual, and the type of support provided by healthcare providers, which prevented us from drawing more detailed conclusions regarding these aspects. The articles published before 2018 did not address new technologies as we defined and considered them in our review. This lack of inclusion of significant technological interventions in older studies limited our ability to analyze these resources in the context of our research, as they did not provide relevant data on the use and effectiveness of new technologies in supporting family caregivers. This review was based on a search conducted in PubMed, Scopus, and CINAHL, which may have limited the breadth of our findings due to the reliance on a restricted set of databases. Lastly, by not focusing the analysis on a specific disease or group of diseases, we provide an overall view of the situation and facilitate hypothesis generation; nonetheless, a focus on a specific pathology would have improved the coherence of the results and allowed for more precise conclusions.

In conclusion, the reviewed studies identify several common barriers faced by caregivers, such as lack of digital literacy, connectivity issues, and resistance to using new technologies, especially in older populations. Suggested solutions include personalized follow-up sessions and training in the use of technologies. On the other hand, the pros of these interventions include improvements in caregivers’ mental health, reductions in depressive symptoms, and increased resilience, while the cons may include a lack of personalization in some programs and limitations in the generalization of results due to the diversity of contexts and populations. Identifying and addressing these issues is essential to build a more effective future for technological support interventions aimed at caregivers of dependent people.

## 5. Conclusions

This study demonstrates that the technological interventions most commonly implemented are online sessions via videoconference, which have shown promising results in improving the mental health of family caregivers by reducing depressive symptoms, stress, and caregiver overload. However, challenges related to connectivity and digital literacy remain significant obstacles. While most of the family caregivers included in the studies were responsible for individuals with some form of dementia, the broad variability in inclusion criteria underscores the importance of tailoring interventions to the specific needs of both caregivers and care recipients to maximize their effectiveness. The importance of continuous follow-up to evaluate and improve interventions over the long term is also emphasized. These findings highlight the need to continue developing support policies that consider both technological preferences and individual circumstances of caregivers.

This study highlights not only the positive aspects of technological interventions but also the observed limitations. Although online sessions have been shown to improve caregivers’ mental health, it is essential to recognize that the effectiveness of these interventions may be affected by a lack of connectivity and digital literacy. Therefore, it is imperative to promote policies that address both technological barriers and the specific needs of caregivers, personalizing interventions to maximize benefits. This proactive approach will not only improve caregivers’ well-being but also ensure that the individuals they care for receive the attention they need.

## Figures and Tables

**Table 1:** Search strategy.

Database	String	Date	Results
CINAHL	“Family caregivers” AND (Interventions OR strategies OR “best practice” OR treatment OR therapy OR program OR management) AND (telehealth OR telemedicine OR telemonitoring OR telepractice OR telenursing OR telecare) NOT (“cancer patients” OR “oncology patients” OR “Patients with cancer” OR palliative) NOT (“mental health” OR “mental illness” OR “mental disorder” OR “psychiatric illness”) NOT (children OR adolescents OR youth OR child OR teenager).	30 January 2024	106
Scopus	(TITLE-ABS-KEY(“Family caregivers”) AND TITLE-ABS-KEY((Interventions OR strategies OR “best practice” OR treatment OR therapy OR program OR management)) AND TITLE-ABS-KEY((telehealth OR telemedicine OR telemonitoring OR telepractice OR telenursing OR telecare)) AND NOT TITLE-ABS-KEY((“cancer patients” OR “oncology patients” OR “Patients with cancer” OR palliative)) AND NOT TITLE-ABS-KEY((“mental health” OR “mental illness” OR “mental disorder” OR “psychiatric illness”)) AND NOT TITLE-ABS-KEY((children OR adolescents OR youth OR child OR teenager)))	30 January 2024	67
PubMed	(“family caregivers”) AND (interventions OR strategies OR “best practice” OR treatment OR therapy OR program OR management) AND (telehealth OR telemedicine OR telemonitoring OR telepractice OR telenursing OR telecare) NOT (“cancer patients” OR “oncology patients” OR “patients with cancer” OR palliative) NOT (“mental health” OR “mental illness” OR “mental disorder” OR “psychiatric illness”) NOT (children OR adolescent OR youth OR child OR teenager)	30 January 2024	116

**Table 2:** Summary of the articles included in the scoping review.

Study Code	Title	Author/Year	Country	Sample Size	Study Methods	Intervention	Results
S1	Acceptance and Commitment Therapy (ACT) Guided Online for Distressed Caregivers of Persons Living with Dementia.	Han et al., 2022 [10]	USA	N = 7	Prospective Study	Online video conference sessions	Reduces anxiety and overload; helps caregivers take better care of themselves.
S2	Effects of a family-focused dyadic psychoeducational intervention for stroke survivors and their family caregivers: a pilot study.	Mou et al., 2022 [11]	China	N = 40	Randomized Clinical Trial	In-person and telephone sessions	Low interest in the digital tool. Preference for in-person sessions. Differences in outcomes based on generation.
S3	Evaluating the efficacy of TeleFAMILIES: a telehealth intervention for caregivers of community-dwelling people with dementia.	Rice et al., 2022 [12]	USA	N = 216	Non-randomized Trial	Online sessions, videoconferencing	Reduces caregiver overload and depressive risk. Increases interaction with other caregivers.
S4	Feasibility and Acceptability of a Remotely Delivered Weighted Blanket Intervention for People Living with Dementia and Their Family Caregivers.	Harris and Titler, 2022 [13]	USA	N = 21	Prospective Study	Online, videoconference, and telephone sessions	Reduces symptoms of anxiety and improves sleep quality. Increases the sense of relaxation.
S5	Increasing Caregiver Access to Programming: A Qualitative Exploration of Caregivers’ Experience of a Telehealth Powerful Tools for Caregivers Program.	Serwe et al., 2019 [14]	USA	N = 12	Qualitative Study	Online sessions, videoconferencing	Caregivers gain knowledge about relaxation techniques and self-care. Increases connection with other caregivers.
S6	Lessons learned from the implementation of a video health coaching technology intervention to improve self-care of family caregivers of adults with heart failure.	Hirschman et al., 2021 [15]	USA	N = 250	Randomized Clinical Trial	Online sessions, videoconferencing	There were various connectivity issues in conducting the online sessions. Alternatives (such as telephone intervention) are necessary.
S7	Results of a Randomized Trial Testing the Efficacy of Tele-Savvy, an Online Synchronous/Asynchronous Psychoeducation Program for Family Caregivers of Persons Living with Dementia.	Hepburn et al., 2022 [16]	USA	N = 261	Randomized Longitudinal Cohort Study	Online sessions, videoconferencing	Improves caregivers’ knowledge and emotional well-being. These benefits may be temporary and decrease over time.
S8	The impact of a pilot telehealth coaching intervention to improve caregiver stress and well-being and to increase dietary protein intake of caregivers and their family members with dementia—Interrupted by COVID-19.	D’Avolio et al., 2023 [17]	USA	N = 25	Randomized Clinical Trial	Online sessions, videoconferencing	Improves support for caregivers. Enhances dietary intake among caregivers and those in their care. No reduction in overload or stress is observed.
S9	Determining Evidence for Family Caregiver Communication: Associating Communication Behaviors with Breakdown and Repair.	Williams et al., 2023 [18]	USA	N = 53	Randomized Clinical Trial	Video recordings for sequential behavior analysis	Effective communication strategies between the caregiver and the person with dementia have been identified. It improves training in communication for family caregivers.
S10	The Effect of Education through Telenursing on the Caregiver Burden among Family Caregivers of COVID-19 Patients: A Randomized Clinical Trial.	Rad et al., 2023 [19]	Iran	N = 66	Randomized Clinical Trial	Tele-nursing education sessions	Reduces caregiver overload. Enhances caregiver training.
S11	IN-HOME-PD Caregivers: The effects of a combined home visit and peer mentoring intervention for caregivers of homebound individuals with advanced Parkinson’s disease.	Fleisher et al., 2023 [20]	USA	N = 65	Non-randomized Controlled Trial	In-person home visits, online sessions (videoconferences), telephone sessions	There was no reduction in caregiver overload. Caregivers were satisfied with both the home visits and the online sessions conducted by the interdisciplinary team.
S12	‘Now I can bend and meet people virtually in my home’: The experience of a remotely supervised online chair yoga intervention and visual socialisation among older adults with dementia.	Park et al., 2023 [21]	USA	N = 9	Qualitative Study	Online yoga sessions, follow-up videoconference	Highlights the convenience and follow-up in the caregiver’s yoga practice and virtual socialization.
S13	Psychological Support for Family Caregivers of Patients with Amyotrophic Lateral Sclerosis at the Time of the Coronavirus Disease 2019 Pandemic: A Pilot Study Using a Telemedicine Approach.	Sharbafshaaer et al., 2022 [22]	Italy	N = 12	Randomized Controlled Pilot Study	Online sessions, videoconferences, and follow-up telephone sessions	No differences are observed in the reduction of caregiver overload and stress. Increases caregivers’ knowledge of the disease.
S14	Hear–Communicate–Remember: Feasibility of delivering an integrated intervention for family caregivers of people with dementia and hearing impairment via telehealth.	Meyer et al., 2020 [23]	Australia	N = 6	Qualitative Study	Online sessions, videoconferencing, and video viewing	Improves knowledge about the use of hearing aids and communication between the person receiving care; as well as the psychosocial well-being of caregivers, but there is a lack of familiarity with the technology.
S15	Pilot Test of a Computer-Based System to Help Family Caregivers of Dementia Patients.	Gustafson et al., 2019 [24]	USA	N = 31	Pilot Study	Online platform for tools and resources, and videoconferences	No improvements are observed in caregiver overload or depressive symptoms, but there is an improvement in coping and a slight improvement in anxiety. Social support improves.
S16	Efficacy of Internet-Delivered Mindfulness for Improving Depression in Caregivers of People with Spinal Cord Injuries and Chronic Neuropathic Pain: A Randomized Controlled Feasibility Trial.	Hearn et al., 2019 [25]	UK	N = 55	Randomized Clinical Trial	Online mindfulness training course	High adherence rate to the program. There was a reduction in depressive symptoms and anxiety, along with improvements in psychological and social quality of life.
S17	Family Health Conversations Conducted by Telephone in Heart Failure Nursing Care: A Feasibility Study.	Gusdal et al., 2018 [26]	Sweden	N = 8	Single-group Intervention Study	Telephone sessions on family health conversations	Improves relationships between family and nursing, and within the family. Nurses gain more knowledge about the family.
S18	Home-Based Video Telemedicine for Dementia Management.	Moo et al., 2020 [27]	USA	N = 222	Retrospective Cross-sectional Study	Online sessions, videoconferencing	Similar satisfaction between in-person sessions and telemedicine, although slightly higher with the latter. Greater acceptance among younger individuals.
S19	Supporting Family Caregivers with Technology for Dementia Home Care: A Randomized Controlled Trial.	Williams et al., 2019 [28]	USA	N = 154	Randomized Clinical Trial	Telehealth sessions and personalized video recordings	Reduces caregiver overload, depressive symptoms, sleep disturbances, and desire for institutionalization. Improves knowledge of the disease.
S20	Enhancing Resilience in Family Caregivers Using an mHealth App.	Smealie et al., 2022 [29]	USA	N = 30	Qualitative Study	App with various tools, resources, tracking, and forums	Ease of use of the app. The app helped foster resilience by promoting positive emotions and self-care practices.
S21	Value co-creation with family caregivers to people with dementia through a tailor-made mHealth application: a qualitative study.	Kagwa et al., 2022 [30]	Sweden	N = 12	Qualitative Study	Use of a caregiver support app. Resources and tools for caregiver self-care.	Increased access to health services and personalized information. Empowerment in decision-making and caregiving capacity. Reduces caregiver overload.
S22	Caregiver Satisfaction with a Video Telehealth Home Safety Evaluation for Dementia.	Gately et al., 2020 [31]	USA	N = 10	Mixed Methods	Home safety assessment via video in telehealth	Overall positive satisfaction among caregivers with the use of video telehealth. It is necessary to address technological issues.
S23	Co-creation of mHealth intervention for older adults with hip fracture and family caregivers: a qualitative study.	Ariza-Vega et al., 2024 [32]	Spain	N = 21	Qualitative Study	Use of a mobile app for caregiver support with resources and tools	The app needs to have a simple and user-friendly design. The information was relevant regarding recovery, prevention, and personalized exercises.
S24	Using Technology to Facilitate Fidelity Assessments: The Tele-STAR Caregiver Intervention.	Lindauer et al., 2019 [33]	USA	N = 13	Pilot Study	Online sessions, videoconferencing with nursing consultation	Reduction in caregiver overload and problem-solving in care. Most consider the intervention convenient, preferring it over in-person sessions.
S25	The perceived quality of video consultations in geriatric outpatient care by early adopters.	Spronk et al., 2022 [34]	Netherlands	N = 7	Qualitative Study	Video consultations	Technological issues causing slow implementation.
S26	Effect of Telenursing on Levels of Depression and Anxiety in Caregivers of Patients with Stroke: A Randomized Clinical Trial.	Goudarzian et al., 2018 [35]	Iran	N = 152	Randomized Clinical Trial	Telephone and online sessions	Reduction of anxiety and depressive symptoms in the caregiver.
S27	The Journey of Recovery: Caregivers’ Perspectives from a Hip Fracture Telerehabilitation Clinical Trial.	Ariza-Vega et al., 2021 [36]	Spain	N = 44	Descriptive Study	Online session, tele-rehabilitation	Improves recovery at home and provides caregivers with knowledge for managing care at home. Levels of stress and anxiety were reduced.
S28	Self-Management Support and eHealth When Managing Changes in Behavior and Mood of a Relative with Dementia: An Asynchronous Online Focus Group Study of Family Caregivers’ Needs.	Huis in Het Veld et al., 2018 [37]	Netherlands	N = 32	Descriptive Study	Asynchronous online debates in discussion groups	Regarding eHealth, participants felt that it can be useful but does not fit every personal situation.
S29	The feasibility and preliminary effects of a pilot randomized controlled trial: Videoconferencing acceptance and commitment therapy in distressed family caregivers of people with dementia.	Han et al., 2023 [38]	USA	N = 19	Randomized Clinical Trial	Sessions via video calls and psychoeducational materials	Improvement in acceptance of the illness. Psychological distress and quality of life in caregivers also improved.
S30	Perceptions and Attitudes toward a Proposed Digital Health Physical Activity Program among Older Family Caregivers of Persons with Heart Failure: A Qualitative Study.	Baik et al., 2023 [39]	USA	N = 13	Qualitative Study	Physical activity sessions via videoconference (Zoom), activity tracker (Fitbit), and motivational text messages.	Positive experience with technology, good digital skills, but issues with internet connectivity for videoconferences.
S31	Effects of a Video-based Intervention on Caregiver Confidence for Managing Dementia Care Challenges: Findings from the FamTechCare Clinical Trial.	Shaw et al., 2020 [40]	USA	N = 84	Randomized Clinical Trial	Online, videoconference, and telephone sessions	There are no significant differences.
S32	“It Took the Stress out of Getting Help”: The STAR-C-Telemedicine Mixed Methods Pilot.	Lindauer et al., 2018 [41]	USA	N = 20	Mixed Methods	Videoconference sessions	Reduction in caregiver overload. Cost savings compared to traditional programs.

**Table 3:** Types of interventions by country of publication.

Study Code	Intervention	Country
S1, S3, S5, S6, S7, S8, S18, S32	Online video conference sessions	USA
S4, S31	Online, videoconference, and telephone sessions	
S9	Video recordings for sequential behavior analysis	
S11	In-person home visits, online sessions (videoconferences), and telephone sessions	
S12	Online yoga sessions, follow-up videoconference	
S15	Online platform for tools and resources, and videoconferences	
S19	Telehealth sessions and personalized video recordings	
S20	App with various tools, resources, tracking, and forums	
S22	Home safety assessment via video in telehealth	
S24	Online sessions, videoconferencing with nursing consultation	
S29	Sessions via video calls and psychoeducational materials	
S30	Physical activity sessions via videoconference (Zoom), activity tracker (Fitbit), and motivational text messages	
S2	In-person and telephone sessions	ASIA: China Iran
S10	Tele-nursing education Sessions	
S26	Telephone and online sessions	
S13	Online sessions, videoconferences, and follow-up telephone sessions	EUROPE: Italy UK Sweden Spain Netherlands
S16	Online mindfulness training course	
S17	Telephone sessions on family health conversations	
S21, S23	Use of a caregiver support app. Resources and tools for caregiver self-care	
S25	Video consultations	
S27	Online session, tele-rehabilitation	
S28	Asynchronous online debates in discussion groups	
S14	Online sessions, videoconferencing, and video viewing	Australia

**Table 4:** Duration of the interventions in the reviewed articles.

Article	Intervention Duration	Percentage
S9	1 day	3.13%
S23	3 days	3.13%
S22	5 days	3.13%
S28	2 week	3.13%
S2, S4, S10, S14	4 week	12.25%
S5, S7	6 week	6.25%
S8, S12, S16, S17, S21, S24, S29, S32	8 week	25%
S1	10 week	3.13%
S13, S19, S26, S27, S31	3 months	15.63%
S20, S30	4 months	6.25%
S25	5 months	3.13%
S3, S6, S15	6 months	9.38%
S18	9 months	3.13%
S11	1 year	3.13%

## Data Availability

Data are available from the investigators on reasonable request. Requests should be directed to the corresponding author by email.

## Supplementary Materials

The following supporting information can be downloaded at https://www.mdpi.com/article/10.3390/healthcare12232350/s1, Table S1: PRISMA-ScR checklist.
